# *Lrig1* is a haploinsufficient tumor suppressor gene in malignant glioma

**DOI:** 10.1038/s41389-017-0012-8

**Published:** 2018-02-02

**Authors:** Feng Mao, Camilla Holmlund, Mahmood Faraz, Wanzhong Wang, Tommy Bergenheim, Samuel Kvarnbrink, Mikael Johansson, Roger Henriksson, Håkan Hedman

**Affiliations:** 1 Department of Radiation Sciences, Oncology, Umeå University, Umeå, Sweden; 2 Department of Pathology/Cytology, Karolinska University Hospital, Huddinge, Sweden; 3 Department of Pharmacology and Clinical Neuroscience, Section of Neurosurgery, Umeå University, Umeå, Sweden; 4 Regionalt Cancercentrum Stockholm Gotland, Karolinska, Stockholm, Sweden; 5 Present Address: Department of Neurosurgery, Tongji Hospital, Tongji Medical College, Huazhong University of Science and Technology, Wuhan, China

## Abstract

Recently, a genome-wide association study showed that a single nucleotide polymorphism (SNP) —*rs11706832*—in intron 2 of the human *LRIG1* (*Leucine-rich repeats and immunoglobulin-like domains 1*) gene is associated with susceptibility to glioma. However, the mechanism by which *rs11706832* affects glioma risk remains unknown; additionally, it is unknown whether the expression levels of *LRIG1* are a relevant determinant of gliomagenesis. Here, we investigated the role of *Lrig1* in platelet-derived growth factor (PDGF)-induced experimental glioma in mice by introducing mono-allelic and bi-allelic deletions of *Lrig1* followed by inducing gliomagenesis via intracranial retroviral transduction of *PDGFB* in neural progenitor cells. *Lrig1* was expressed in *PDGFB*-induced gliomas in wild-type mice as assessed using in situ hybridization. Intriguingly, *Lrig1*-heterozygous mice developed higher grade gliomas than did wild-type mice (grade IV vs. grade II/III, *p* = 0.002). Reciprocally, the ectopic expression of LRIG1 in the TB107 high-grade human glioma (glioblastoma, grade IV) cell line decreased the invasion of orthotopic tumors in immunocompromised mice in vivo and reduced cell migration in vitro. Concomitantly, the activity of the receptor tyrosine kinase MET was downregulated, which partially explained the reduction in cell migration. In summary, *Lrig1* is a haploinsufficient suppressor of PDGFB-driven glioma, possibly in part via negative regulation of MET-driven cell migration and invasion. Thus, for the first time, changes in physiological Lrig1 expression have been linked to gliomagenesis, whereby the SNP *rs11706832* may affect glioma risk by regulating LRIG1 expression.

## Introduction

Both adult and pediatric diffuse gliomas are devastating diseases with considerable morbidity and poor cure rates. Although these diseases share clinical and molecular features, they are also distinct. Gliomas frequently display dysregulated growth factor signaling; it is estimated that 88% of adult glioblastomas harbor mutations that affect the receptor tyrosine kinase (RTK)/RAS/PI(3)K signaling axis^1^. For example, 35% of proneural glioblastomas have focal amplification of *PDGFRA* (which encodes platelet-derived growth factor receptor alpha, PDGFRA) whereas 11% have mutations in *PDGFRA*; 97% of classical glioblastomas have high-level *EGFR* (encoding epidermal growth factor receptor, EGFR) amplification whereas 55% have mutations in *EGFR*; and 37% of mesenchymal glioblastomas have a deletion of *NF1* (encoding neurofibromin 1, NF1)^2^. Indeed, the *PDGFA* gene was recently shown to drive all non-CpG island methylator phenotype glioblastomas^3^. In contrast to adult diffuse gliomas, which typically originate in cerebral white matter, most pediatric diffuse gliomas arise in the pons region of the brainstem (diffuse intrinsic pontine glioma, DIPG)^4^. The mutational profiles of DIPGs are different from those of adult diffuse gliomas. For example, DIPGs frequently harbor mutations in histone H3 (typically p.Lys27Met mutations in *H3F3A* or *HIST1H3B* in 88% of cases)^5^ and activating mutations in *ACVR1* (encoding ACVR1/ALK4, a bone morphogenetic protein type I receptor; 20–32% of cases)^5–7^. However, similar to many adult diffuse gliomas, DIPGs frequently show focal gains in *PDGFRA* (36–40% of cases)^6,8^, including activating point mutations of the gene (5% of DIPG cases)^9^. Thus, although there are important differences in diffuse gliomas between adults and children, the frequent activation of growth factor receptor signaling in general, particularly PDGFR signaling, appears to be common in gliomas that manifest in both populations.

Recently, a genome-wide association study revealed that a single-nucleotide polymorphism (SNP) in intron 2 of *LRIG1* (encoding leucine-rich repeats and immunoglobulin-like domains (LRIG)-1) influences the risk of occurrence of diffuse glioma^10^. LRIG1 is an integral membrane protein belonging to the LRIG family^11–13^ and negatively regulates various oncogenic RTKs, including EGFR^14,15^, EGFRvIII^16^, hepatocyte growth factor receptor (MET)^17^, RET proto-oncogene product (RET)^18^, and PDGFRA^19^. *Lrig1* knockout mice have hyperproliferative epidermal and intestinal stem cells^20–23^. Moreover, LRIG1 expression is a good prognostic indicator of a variety of human cancers^24^. The non-physiological overexpression of LRIG1 has been shown to inhibit the proliferation of certain glioma cell lines in vitro^16,25,26^. Additionally, a soluble form of the LRIG1 ectodomain can inhibit EGFR signaling in trans as well as suppress the proliferation of glioma cells in vitro and the growth of human glioma xenografts in vivo^26–28^. However, the physiological role of *LRIG1* in gliomagenesis has not been experimentally investigated to date.

In the present study, we investigated the role of physiological *Lrig1* expression in PDGFB-induced glioma in mice and analyzed the effects of forced LRIG1 overexpression on human glioblastoma xenografts in vivo and on human glioblastoma cells in vitro.

## Results

### *Lrig1* was expressed in PDGFB-induced mouse gliomas

**Fig. 1:**
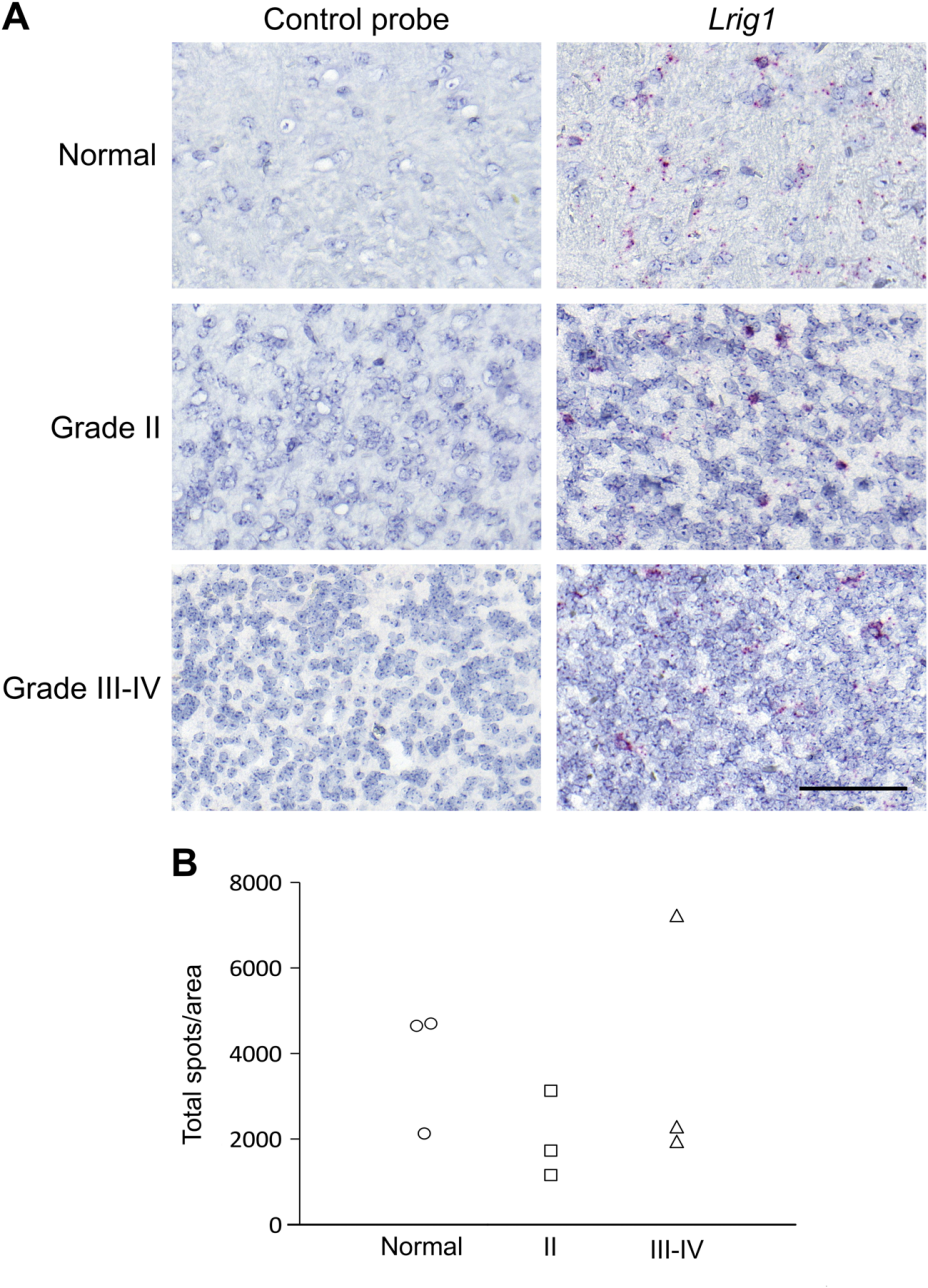
In situ hybridization of *Lrig1* in normal brain tissue and PDGFB-induced glioma in mice Newborn *Ntv-a* mice were transduced with PDGFB-expressing avian retroviruses. At 12 weeks of age, the mice were sacrificed, and their brains were dissected and analyzed via in situ hybridization. **a** Micrographs showing images of in situ hybridized sections of normal brain (upper row), grade II glioma (middle row), and grade III–IV glioma (lower row). The left panel shows sections hybridized with the negative control probe, whereas the right panel shows sections hybridized with the *Lrig1* probe. Scale bar, 100 μm. **b** Quantification of the total number of signals (single spots and clusters) per area in normal brain (*n* = 3), grade II glioma (*n* = 3), and grade III–IV glioma (*n* = 3). There was no significant difference in *Lrig1* expression (in situ hybridization signals per area) among the groups

To address the role of LRIG1 in PDGF-driven gliomas, we used the RCAS/Ntv-a system to induce glioma in mice with different *Lrig1* genotypes via the intracranial transduction of neural progenitor cells with PDGFB-encoding RCAS viruses. In this glioma model, most PDGFB-transduced mice develop gliomas within 12 weeks of age; these lesions present either oligodendroglial or glioblastoma-like morphology^19,29^. We have previously shown that these tumors express *Lrig2*^19^. To investigate whether these tumors also express *Lrig1*, we performed in situ hybridization (Fig. 1a) and observed that the PDGFB-induced gliomas as well as normal mouse brain tissue expressed *Lrig1*. Quantification of the in situ hybridization signals for *Lrig1* from three normal brains, three grade II–III tumors, and three grade IV tumors showed pronounced intra-group variability and a lack of a consistent difference among the groups (Fig. 1b).

### Generation and characterization of *Lrig1*-deficient *Ntv-a* mice

**Fig. 2:**
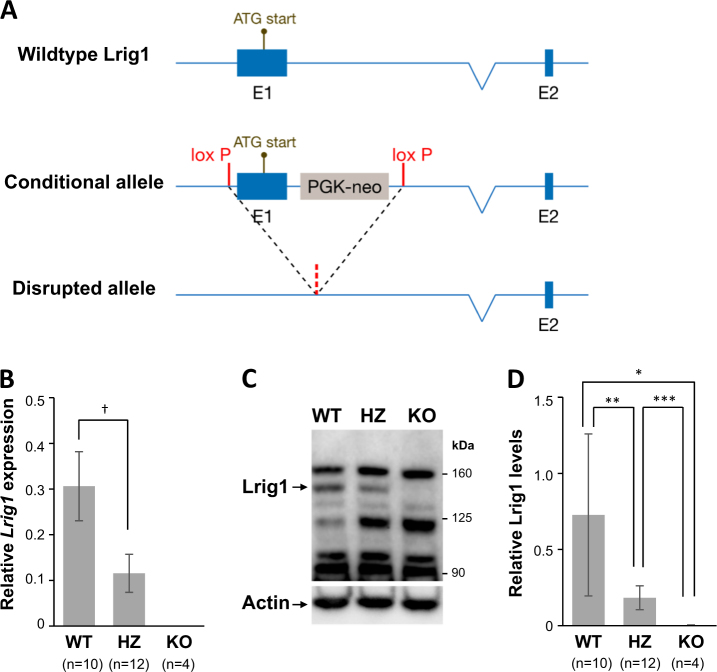
Generation and characterization of *Lrig1*-deficient mice **a** Schematic representation of wild-type, conditional knockout, and disrupted *Lrig1* alleles. A PKG-neo selection cassette was inserted downstream of exon 1 of *Lrig1*. Both exon 1 and the PKG-neo cassette were flanked by loxP sites; thus, both regions could be deleted in a single step by mating the mice with OzCre mice to generate the disrupted *Lrig1* allele. **b** Relative *Lrig1* mRNA expression levels in mouse brains from wild-type (WT), *Lrig1*-heterozygous (HZ), and *Lrig1*-deficient (KO) mice. Total RNA was prepared from the brains of 1-week-old mice, and the *Lrig1* expression levels were determined with quantitative RT-PCR. The *Lrig1* levels were normalized to those of the internal control gene *Rn18s*. An arbitrary scale was established such that the mean *Lrig1* levels in the mouse reference RNA had a value 1. In the *Lrig1*-deficient mice, *Lrig1* could not be detected in any of the samples analyzed. Error bars indicate ±standard deviations. †*p* = 6.6 × 10^−7^, Student’s *t*-test. **c** Western blot analysis of brain lysates from 1-week-old wild-type (WT), *Lrig1*-heterozygous (HZ), and *Lrig1*-deficient (KO) mice using antibodies against Lrig1 or actin. **d** Quantification of Western blots of brain lysates from wild-type mice, *Lrig1*-heterozygous mice, and *Lrig1*-knockout mice. The mean Lrig1/actin ratios are shown on an arbitrary scale. Error bars indicate ±standard deviations. *p = 0.025, **p = 0.0045, and ***p = 0.00088, Student’s *t*-test

To further explore the role of *Lrig1* in PDGFB-driven malignant gliomas, *Lrig1*-deficient mice were generated by knocking out exon 1 of *Lrig1* (Fig. 2a). The *Lrig1*-deficient mice (B6.129-*Lrig1*^*tm1.1Hhed*^) showed no signs of illness or any other overt phenotype other than psoriasis-like lesions on their tails as previously described in another *Lrig1*-deficient mouse strain^20^. The quantitative reverse transcription polymerase chain reaction (RT-PCR) results demonstrated that compared to the wild-type mice, 1-week-old *Lrig1*-deficient mice presented no detectable *Lrig1* transcripts in their brains, and mice heterozygous for this deficiency presented reduced levels (Fig. 2b). The Western blot results confirmed the complete absence of full-length Lrig1 protein in the brains of *Lrig1*-deficient mice and reduced levels in heterozygous mice compared to the levels in wild-type mice (Fig. 2c, d).

### Malignancy of PDGFB-induced gliomas was enhanced by the ablation of one *Lrig1* allele

**Fig. 3:**
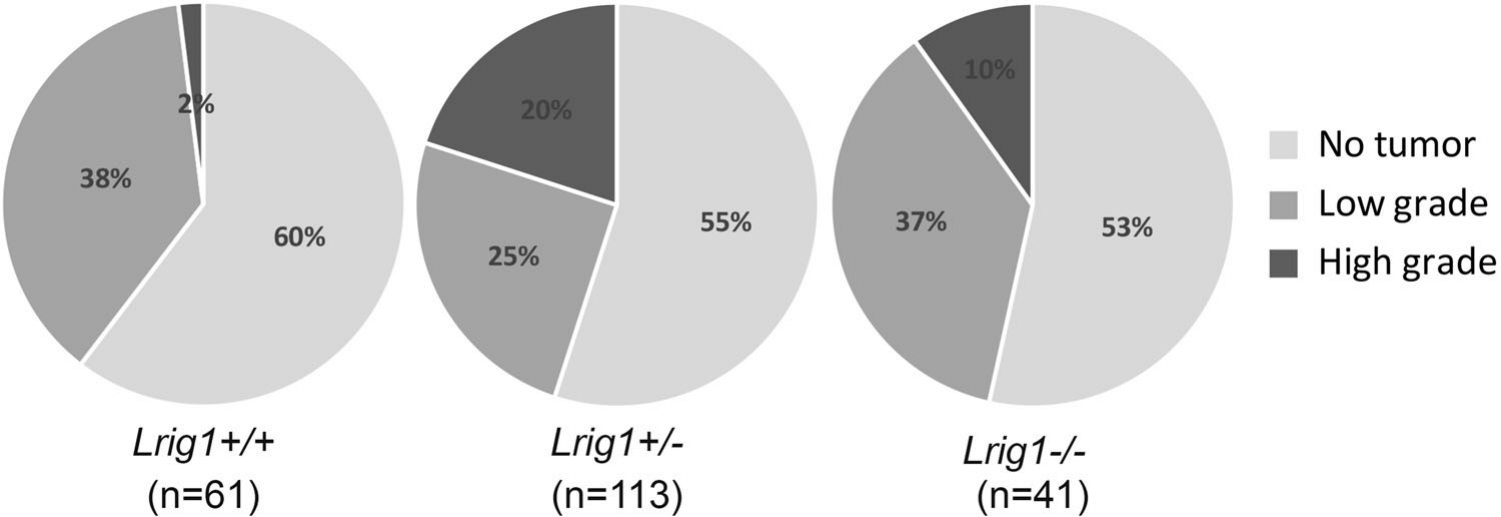
The incidence of PDGFB-induced glioma according to *Lrig1* genotype and histological grade Newborn mice with different *Lrig1* genotypes were transduced with PDGFB-encoding RCAS retroviruses. At 12 weeks of age, the mice were sacrificed, and their brains were examined. Circles show no tumors (light gray), low-grade tumors (grade II, intermediate gray), and high-grade tumors (grade III–IV, dark gray) according to *Lrig1* genotype; wild-type (*Lrig1+/+*, *n* = 61), *Lrig1*-heterozygous (*Lrig1+/−*, *n* = 113), and *Lrig1*-deficient (*Lrig1−/−*, *n* = 41). The *Lrig1*-heterozygous mice developed significantly more high-grade tumors than did wild-type mice (Pearson’s *χ*^2^ test, *p* = 0.002)

The *Lrig1*-deficient mouse strain was crossed with transgenic *Ntv-a* mice followed by backcrossing of those offspring with the *Ntv-a* mice; this husbandry setup generated mice homozygous for *Ntv-a* and heterozygous for *Lrig1* (*Ntv-a+/+*; *Lrig1+/−*). These mice were intercrossed, and their offspring were injected with PDGFB-encoding RCAS virus-producing DF-1 cells at birth and analyzed at 12 weeks of age. The mice developed no tumors, low-grade tumors (grade II–III), or glioblastoma-like high-grade tumors (grade IV) (Fig. 3). The overall tumor incidence was similar among the genotypes; however, the incidence of high-grade tumors was significantly higher in the *Lrig1*-heterozygous mice than that in the wild-type mice (19.6 vs. 1.6%, respectively; Pearson’s test, *p* = 0.002). The differences in the incidence of low-grade and high-grade tumors between the wild-type and knockout mice or between the heterozygous and knockout mice were not statistically significant (Pearson’s *χ*^2^ test; *p* = 0.173 and *p* = 0.204, respectively).

### Invasion of TB107-derived malignant glioma was suppressed by LRIG1 overexpression in vivo

**Fig. 4:**
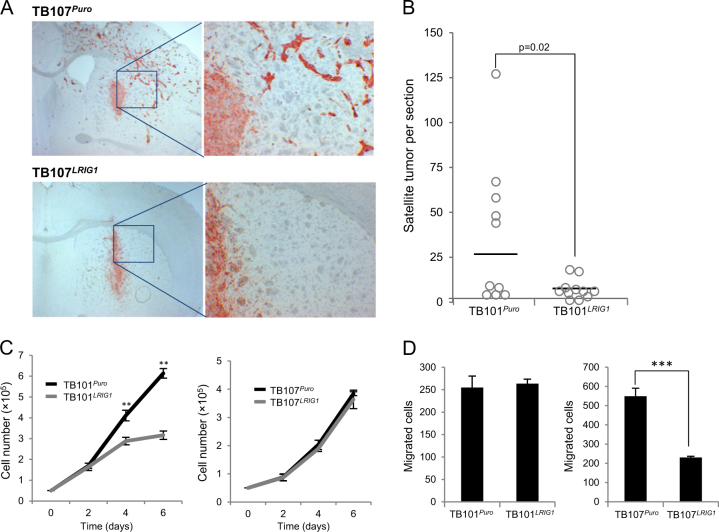
Effects of LRIG1 expression on the invasion, proliferation, and migration of glioma cells TB101 and TB107 cells were transduced with either control vector, to produce TB101^*Puro*^ and TB107^*Puro*^ cells or a stable *LRIG1* expression vector to produce TB101^*LRIG1*^ and TB107^*LRIG1*^ cells. **a** Nude mice were intracranially injected with TB107^*Puro*^ or TB107^*LRIG1*^ cells. Four months after injection, the mice were killed, and their brains were analyzed using immunohistochemistry. Micrographs showing representative images of mouse brains transplanted with TB107^*Puro*^ cells or TB107^*LRIG1*^ cells and stained for human vimentin to visualize the tumor cells. **b** Representation showing the quantification of the number of satellite tumors per section from mice with TB107^*Puro*^ (*n* = 10) and TB107^*LRIG1*^ tumors (*n* = 12). Horizontal lines indicate the medians of the respective group. The difference in the number of satellite tumors surrounding the TB107^*Puro*^ and TB107^*LRIG1*^ primary tumors was significant (Student’s *t*-test, *p* = 0.02). **c** Proliferation rates of the respective cell lines. Error bars indicate ± standard deviations of the means from triplicate samples. One of three similar experiments is shown. The difference in proliferation rate between TB101^*Puro*^ and TB101^*LRIG1*^ cells was significant (**p < 0.01, Student’s *t*-test). **d** Migration of the respective cell lines. Cells were seeded in transwell inserts with serum-free medium in the upper chamber and medium containing 5% FBS in the lower chamber. After the cells were incubated 24 h, the inserts were fixed and stained with crystal violet, and the cells that migrated across the membrane were counted. The mean number of migratory cells per ten high-power fields in one of three similar experiments is shown; standard deviations of triplicate samples are indicated with error bars. ***p < 0.001, Student’s *t*-test

Because ablating one copy of *Lrig1* resulted in a decrease of Lrig1 expression and an increase in the tumor grade (grade IV vs. grade II–III), we surmised that there exists a relationship between the reduced expression of Lrig1 and the increased malignancy of tumors. Therefore, we tested the complementary hypothesis, i.e., whether increasing LRIG1 expression in high-grade glioma cells would reduce their malignancy. To this end, we transduced two human high-grade glioblastoma cell lines, TB101 and TB107, with lentiviral vectors expressing either LRIG1 (*pLVX-LRIG1*) or empty control vector (*pLVX-Puro*) and assessed the behavior of the transduced cells in immunocompromised mice in vivo. The transduced cells were orthotopically transplanted into the brains of immunodeficient BALB/cA nude mice. Four months after transplantation, the mice were sacrificed and analyzed. The incidence of tumors among the mice transplanted with TB101^*Puro*^, TB101^*LRIG1*^, TB107^*Puro*^, and TB107^*LRIG1*^ were 11/12, 9/11, 10/10, and 12/12, respectively. Unfortunately, the primary tumors of the mice transplanted with TB101 cells were very small with few satellite colonies; these lesions were difficult to delineate. Thus, no further analyses of the TB101^*Puro*^ and TB101^*LRIG1*^ groups were performed. By contrast, the primary tumors in the mice transplanted with TB107 cells were easily identifiable. LRIG1 overexpression resulted in the significantly reduced invasion of TB107^*LRIG1*^ tumors into the surrounding brain tissue compared to that of the TB107^*Puro*^ tumors (Fig. 4a, b). Thus, the number of tumor satellites was significantly lower in the LRIG1-overexpressing TB107^*LRIG1*^ tumors than that in the TB107^*Puro*^ tumors (Student’s *t*-test, *p* = 0.02). In vitro, compared with the control TB107^*Puro*^ cells, the LRIG1-overexpressing TB107^*LRIG1*^ cells did not exhibit any changes in their proliferation rate (Fig. 4c); however, migration was reduced in the LRIG1-overexpressing TB107^*LRIG1*^ cells (Fig. 4d). Intriguingly, the TB101 groups showed an inverse pattern, i.e., TB101^*LRIG1*^ cells exhibited a lower proliferation rate than the TB101^*Puro*^ cells and an unaltered migration rate (Fig. 4c, d).

### Cell migration was suppressed by ectopic human LRIG1 expression in a context-dependent manner

**Fig. 5:**
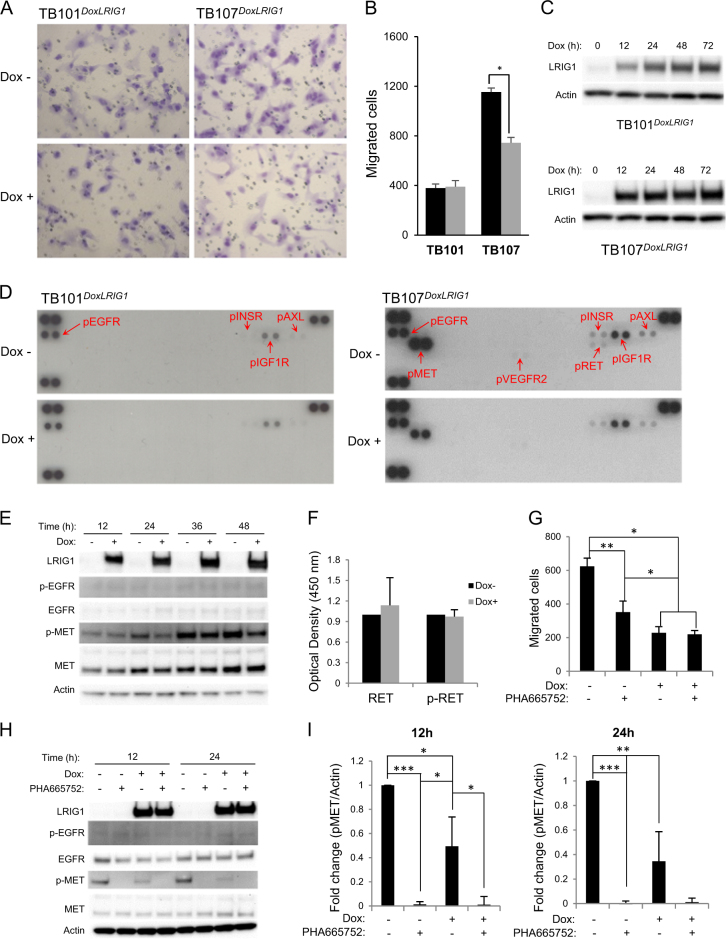
Regulation of glioma cell migration by LRIG1 and the role of RTK phosphorylations **a** TB101^*DoxLRIG1*^ (left column) and TB107^*DoxLRIG1*^ (right column) cells with a doxycycline-inducible *LRIG1* allele were incubated in the presence or absence of doxycycline for 48 h, after which the cells were transferred to transwell inserts and incubated in the presence or absence of doxycycline for an additional 48 h. Micrographs showing representative images of migratory cells incubated in the presence (Dox+) or absence (Dox−) of doxycycline. **b** Bar charts showing the quantifications of the number of migratory cells per five high-power microscopy fields. One of three independent experiments is shown. In each experiment, triplicate wells were analyzed for each treatment, and the error bars indicate standard deviations. *p < 0.05, Student’s *t*-test. **c** Immunoblots showing the induction of LRIG1 by doxycycline at various time points; actin is shown as the loading control. **d** TB101^*DoxLRIG1*^ (left panel) or TB107^*DoxLRIG1*^ (right panel) cells were incubated in the presence (Dox+) or absence (Dox−) of doxycycline for 48 h followed by lysis, and the phospho-RTK levels were analyzed using a phospho-RTK array. The positions and identities of detectable spots are indicated with arrows and include p-EGFR, p-MET, p-INSR, p-IGF1R, p-AXL, p-RET and p-VEGFR2. **e** TB107^*DoxLRIG1*^ cells were incubated in the presence or absence of doxycycline for the indicated times followed by lysis, and an immunoblot analysis for LRIG1, p-EGFR (Tyr1086), total EGFR, p-MET (Tyr1234/1235), and total MET was conducted with actin as the loading control. **f** TB107^*DoxLRIG1*^ cells were incubated in the presence or absence of doxycycline for 48 h followed by lysis, and total RET and p-RET (panTYR) were analyzed using specific ELISA kits. One of three similar experiments depicting the mean of triplicate samples is shown; standard deviations are indicated by error bars. **g–i** Pharmacologic and LRIG1-induced suppression of TB107 cell migration. LRIG1 expression was induced by culturing TB107^*DoxLRIG1*^ cells with doxycycline for 48 h. Then, the cells were seeded in transwell inserts in the presence or absence of the MET inhibitor PHA-665752 for 24 h. To enhance transwell migration via chemotaxis, the medium in the upper chamber was serum-free, whereas the medium in the lower chamber contained 5% FBS. **g** At the end of the experiment, the transwell inserts were fixed and stained with crystal violet, and the number of migratory cells was counted. The mean number of migratory cells per ten high-power fields of three wells from one of three independent experiments is shown. Standard deviations of triplicate wells are indicated by error bars. *p < 0.05 and **p < 0.01, Student’s *t*-test. **h** In parallel experiments, cells were seeded in six-well plates but were otherwise treated in the same manner as the cells in the transwell chambers. After 12 or 24 h of incubation, the cells were lysed and subjected to immunoblot analyses for LRIG1, p-EGFR (Tyr1086), total EGFR, p-MET (Tyr1234/1235), and total MET with actin as the loading control. **i** Quantification of the p-MET/actin ratios 12 h (left) and 24 h (right) after the induction of *LRIG1*. The mean ratios of three independent experiments are shown with standard deviations indicated by error bars. *p < 0.05, **p < 0.01, and ***p < 0.001, Student’s *t*-test

To further analyze the effects of LRIG1 overexpression on the migration of glioma cells, we transduced TB101 and TB107 cells with a doxycycline-inducible *LRIG1* allele and analyzed their migration with or without the induction of ectopic LRIG1 expression. We focused on cell migration because it represents the invasive capacity of tumor cells. The induction of ectopic LRIG1 expression suppressed the migration of TB107^*DoxLRIG1*^ cells but did not affect the migration of TB101^*DoxLRIG1*^ cells (Fig. 5a–c).

### LRIG1 overexpression inhibited the phosphorylation of MET but not of EGFR in TB107 cells

To investigate the mechanism by which LRIG1 overexpression inhibits migration in TB107^*DoxLRIG1*^ cells but not in TB101^*DoxLRIG1*^ cells, we analyzed the levels of phosphorylated RTK (pRTK) using a phospho-RTK array in TB101^*DoxLRIG1*^ and TB107^*DoxLRIG1*^ cells with endogenous or ectopic LRIG1 expression (Fig. 5d). In the non-induced TB101^*DoxLRIG1*^ cells, EGFR, the insulin receptor, IGF1R, and AXL were phosphorylated (Fig. 5d, left, upper panel). Following the induction of LRIG1 overexpression for 48 h, the phosphorylation levels of these proteins remained largely unaffected (Fig. 5d, left, lower panel). In the non-induced TB107 cells, EGFR, MET, the insulin receptor, IGF1R, AXL, RET, and VEGFR2 were phosphorylated (Fig. 5d, right, upper panel). Following the induction of LRIG1 overexpression for 48 h, the phosphorylation of MET and RET was strongly inhibited, whereas the levels of phosphorylated EGFR remained unaffected (Fig. 5d, right, lower panel). To confirm these observations in TB107 cells and to investigate the kinetics of the changes in pRTK levels, immunoblotting and enzyme-linked immunosorbent assay (ELISA) were performed. Immunoblotting confirmed that MET phosphorylation was reduced when LRIG1 was overexpressed, whereas EGFR phosphorylation was not affected (Fig. 5e). The reduction in MET phosphorylation was observed as early as 12 h after the induction of ectopic LRIG1 expression. However, we were unable to confirm any effect of LRIG1 on the phosphorylation of RET using a phosphorylated RET (p-RET)-specific ELISA (Fig. 5f). Based on these data, LRIG1 overexpression inhibits the phosphorylation of MET but not that of EGFR in TB107^*DoxLRIG1*^ cells.

### LRIG1-induced suppression of TB107 cell migration was partially dependent on MET inhibition

Because LRIG1 overexpression reduced phosphorylated MET (p-MET) levels in TB107^*DoxLRIG1*^ cells, we investigated whether LRIG1-induced suppression of TB107^*DoxLRIG1*^ cell migration was mediated through MET inhibition. Migration was analyzed in TB107 cells with endogenous or ectopic LRIG1 expression and in the presence or absence of the MET-specific kinase inhibitor PHA-665752 (Fig. 5g). Both treatment with PHA-665752 and LRIG1 overexpression suppressed the migration of TB107 ^*DoxLRIG1*^ cells; furthermore, the observed suppression of migration was more pronounced in cells with LRIG1 overexpression than in cells treated with PHA-665752. In the former case, the suppressive effect was not further enhanced by treatment with PHA-665752. LRIG1 overexpression partially inhibited MET phosphorylation, whereas 500 nM PHA-665752 completely abolished all detectable MET phosphorylation (Fig. 5h, i). Thus, the migration of TB107^*DoxLRIG1*^ cells was partially dependent on MET activity; however, the LRIG1-mediated suppression of migration of TB107 cells may also involve mechanisms other than those mediated by MET.

## Discussion

Here, we showed that *Lrig1* was a haploinsufficient tumor suppressor of PDGFB-induced experimental diffuse glioma in mice. To the best of our knowledge, this is the first demonstration of haploinsufficiency of any *Lrig* gene. The loss of a functional *Lrig1* allele, which resulted in reduced mRNA and protein expression of Lrig1, promoted the progression of gliomas from grade II/III to grade IV. Our findings are particularly intriguing in the light of the recent demonstration that the SNP *rs11706832* in intron 2 of *LRIG1* constitutes a diffuse glioma susceptibility locus^10^. However, Melin and colleagues did not address the molecular function of *rs11706832* in their study. Therefore, our current demonstration that the LRIG1 expression level is a determinant of the aggressiveness of PDGFB-driven gliomas may be highly relevant to the etiology of human gliomas.

Because the reduced Lrig1 expression increased the malignancy of low-grade diffuse glioma, we tested the complementary hypothesis, i.e., whether forced LRIG1 overexpression in human high-grade glioma cells (i.e., glioblastoma cells) can reduce their malignancy. Accordingly, LRIG1 overexpression in the primary human glioma cell line TB107 suppressed glioma invasion in vivo and cell migration in vitro. In vivo, LRIG1-overexpressing TB107 tumors exhibited a markedly lower intracranial dissemination than the control TB107 tumors. In vitro, the LRIG1-overexpressing TB107 cells did not display any change in the proliferation rate compared with the control cells; however, the LRIG1-overexpressing TB107 cells exhibited reduced migratory activity. Intriguingly, the other human glioblastoma cell line, TB101, exhibited contrary behaviors when LRIG1 was overexpressed, i.e., its migration rate was unaltered whereas its proliferation rate was reduced. Thus, LRIG1 may function as a tumor suppressor in glioma by suppressing cellular invasion and migration as well as cellular proliferation in a cell context-dependent manner.

The LRIG1-mediated suppression of TB107 cell migration was accompanied by a reduction in MET phosphorylation, and treatment with the MET-specific kinase inhibitor PHA-665752 showed that TB107 cell migration was partially dependent on MET kinase activity. This outcome mirrors the results of a previous study, which showed that LRIG1 overexpression inhibited the migration of the breast cancer cell lines MDA-MB-157 and MDA-MB-231, at least in part, by inhibiting MET^30^. Intriguingly, TB101 cells neither expressed detectable phospho-MET, nor exhibited LRIG1-mediated suppression of migration. Although PHA-665752 completely inhibited MET kinase activity in TB107 cells, it could not suppress cell migration to the same degree as LRIG1 overexpression. Furthermore, despite LRIG1 overexpression strongly suppressing TB107 cell migration, this ectopic expression only partially inhibited MET kinase activity. Taken together, these results suggest that LRIG1 suppressed the migration of TB107 cells through a mechanism that is partially dependent on MET inhibition. The molecular mechanisms responsible for MET-independent LRIG1-mediated suppression of migration in TB107 cells still requires elucidation. Nevertheless, our results show that LRIG1 suppresses the migration of glioma cells in a cell context-dependent manner partially via MET inhibition.

LRIG1 has also been reported to negatively regulate RET^18^ and EGFR. However, our results regarding the possible effects of LRIG1 on RET in TB107 cells were ambiguous; the phospho-RTK array showed a reduction in p-RET upon LRIG1 induction, but the p-RET-specific ELISA did not show such a change. Therefore, we could not definitively conclude whether LRIG1 overexpression could suppress RET in TB107 cells. Intriguingly, we did not detect any changes in the phosphorylation levels or protein expression of EGFR upon LRIG1 overexpression in TB107 cells. These results contradict those of several previous studies reporting that LRIG1 negatively regulates EGFR^14–16,22,23,31–33^. However, our inability to detect the inhibition of EGFR phosphorylation in TB107 cells does not exclude the possibility that signaling downstream of EGFR was still impaired by LRIG1 expression. In a previous study, we found that LRIG2 affects transcriptional responses to PDGF stimulation even in the absence of detectable effects on PDGFR phosphorylation^19^.

In summary, our novel finding that *Lrig1* is a haploinsufficient tumor suppressor gene in glioma provides a possible functional link between the SNP *rs11706832* and the etiology of diffuse glioma. More broadly, the results also suggest that other modes of *LRIG1* gene regulation, including promoter methylation, histone acetylation, and gene copy number variations, could be highly relevant in gliomagenesis and glioma progression both in children and in adults. Thus, it will be important to establish whether *rs11706832* truly affects *LRIG1* expression and/or whether *LRIG1* is regulated by other mechanisms in glioma. Additionally, further elucidating the interactions between *LRIG1* and other genes and mutations that regulate the initiation and progression of human diffuse glioma is critical in better understanding the progression of this disease.

## Materials and methods

### Mouse strains and animal husbandry

The housing and care of the mice as well as all experiments were performed in accordance with the European Communities Council Directive (86/609/EEC). The experimental protocols were approved by the Regional Ethics Committee of Umeå University, Umeå, Sweden (registration no. A5-2010, A41-10, and A42-10). The animals were housed under controlled conditions with a 12-h day/night cycle and provided water and standard chow pellets (cat. no. 801730, Special Diets Services, NOVA-SCB Sweden, Sollentuna, Sweden) ad libitum. The construction of the *Lrig1* targeting vector and the generation of mice with floxed (B6.129-*Lrig1*^*tm1Hhed*^) and deleted (B6.129-*Lrig1*^*tm1.1Hhed*^) *Lrig1* alleles were performed at Ozgene (Bentley DC, WA, Australia) using the same protocol as previously described for the generation of the *Lrig2*-deficient mice^19^. A schematic representation of the wild-type, conditional, and knockout *Lrig1* alleles are shown in Fig. 2a. *Ntv-a* transgenic mice that express the Tv-a avian retrovirus receptor under the control of the Nestin (*Ntv-a* mice) promoter were a kind gift from Lene Uhrbom (Uppsala University, Sweden) and Eric Holland (Memorial Sloan-Kettering Cancer Center, NY, USA). Seven-week-old BALB/cA nude mice were obtained from Taconic Europe A/S (Ry, Denmark).

### Retroviral transduction of mice

Newborn mice obtained from cross-breeding *Lrig1*-heterozygous *Ntv-a* mice received an intracranial injection 2 μl (2 × 10^5^ cells) RCAS-PDGFB-HA-producing DF-1 chicken fibroblasts into the right frontal hemisphere with a 10-μl Hamilton syringe as previously described^19^. The mice were euthanized at 12 weeks after injection or earlier if they showed symptoms of disease. The mouse brains were collected, fixed in 4% paraformaldehyde, embedded in paraffin, and analyzed for tumors by a neuropathologist (W.W.) who was blinded to the treatments.

### In situ hybridization

In situ hybridization for *Lrig1* and the control genes was performed using an RNAScope assay (Advanced Cell Diagnostics, Milano, Italy) as previously described^19^. An RNAScope probe set targeting *Lrig1* (probe-Mm-Lrig1, Mouse: 310521) was custom designed by and purchased from Advanced Cell Diagnostics. The RNAScope positive control probe set (probe-Mm-Ppib: 313911) was used to verify the integrity of the RNA, whereas the RNAScope dapB (310043) control probe set was used to assess non-specific signals. The hybridized and stained slides were scanned using a Pannoramic 250 Flash II scanner (3DHistech, Budapest, Hungary) at 400× magnification, and the images were downscaled to 100× and imported into ImageJ software. The in situ hybridization signals were identified via color thresholding with hue = 180–255, saturation = 35–255, and brightness = 0–210, followed by noise reduction using a median filter with radius = 0.5 px. After the signals were dichotomized into single spots or clusters based on a cutoff area of 16 pixels, they were counted. Nuclei were identified via color thresholding with hue = 125–175, saturation = 41–255, and brightness = 0–235, followed by the application of a maximum filter with radius = 1.0 px and a watershed filter. Spots smaller than 16 pixels were excluded.

### Immunoblotting and phospho-RTK assay

Whole-cell lysates were prepared in a modified RIPA buffer as previously described^34^ (Fig. 2) or in a “cell extraction buffer” (Thermo Fisher Scientific, Gothenburg, Sweden) supplemented with either a complete-mini protease inhibitor cocktail or, for phospho-protein analysis, an EDTA-free protease inhibitor cocktail (Roche Diagnostics Scandinavia AB, Bromma, Sweden). Protein concentrations were determined using Pierce BCA Protein Assay Reagent (Thermo Fisher Scientific). Equal amounts of protein were separated via polyacrylamide gel electrophoresis using NuPAGE Novex 3–8% tris-acetate gels (Thermo Fisher Scientific) and electrotransferred onto a polyvinylidene difluoride membrane (Thermo Fisher Scientific). The membrane was blocked with 5% fetal bovine serum (FBS) for phospho-protein analysis and with 5% non-fat milk for all other analyses. Thereafter, the membrane was incubated with primary antibody, washed, and incubated with horseradish peroxidase-conjugated secondary antibody. Immunoreactive proteins were detected and quantified using chemiluminescence with an Amersham ECL Select Western blotting detection reagent (GE Healthcare, Uppsala, Sweden) and a ChemiDoc XRS imaging system with its associated Image Lab software (Bio-Rad Laboratories AB, Solna, Sweden). To screen for changes in phospho-RTK levels, samples containing 150 μg of whole-cell lysates were analyzed using a Human Phospho-RTK Array kit (R&D Systems Europe Ltd., Abingdon, UK) according to the manufacturer’s instructions.

### RNA extraction and quantitative real-time RT-PCR

RNA was prepared from tissues using an RNAqueos kit (Thermo Fisher Scientific) followed by digestion of contaminating DNA using a TURBO DNA-free kit (Thermo Fisher Scientific) according to the manufacturer’s instructions. Quantitative RT-PCR was performed as previously described^13^. Triplicate samples of 20 ng of total RNA were analyzed using a qScript 1-Step qRT-PCR Kit (Quanta Biosciences, Gaithersburg, MD, USA) according to the manufacturer’s instructions on a Bio-Rad CFX96 apparatus (Bio-Rad Laboratories AB). A *Lrig1*-specific TaqMan gene expression assay kit Mm00456116_m1 was obtained from Applied Biosystems (Thermo Fisher Scientific). *Rn18s* (also known as *18 S rRNA)* was used as the internal control for normalization of the *Lrig1* expression data. The *Rn18s*-normalized *Lrig1* levels were divided by the corresponding level obtained from qRT-PCR amplification of QPCR Mouse Reference Total RNA (Agilent Technologies, Santa Clara, CA, USA); the *Lrig1* level in the reference RNA was set to a value of 1.

### Cell culture

Cells were grown in Dulbecco’s modified Eagle’s medium containing 10% FBS and supplemented with 50 µg/ml gentamicin, MEM non-essential amino acids, and 50 µM 2-mercaptoethanol and cultured at 37 °C in an environment containing 5% CO_2_. The cell culture reagents were obtained from Gibco (Thermo Fisher Scientific). The glioma cell line TB101 and its culture conditions have been previously described^35^. The primary glioma cell line TB107 was established from a primary human glioblastoma specimen as previously described for TB101^35^. The TB101 and TB107 cell lines were obtained from glioblastoma patients with informed consent, and the study was approved by the local ethics committee. Lenti-X 293 cells were obtained from Clontech Laboratories, Inc. (Mountain View, CA, USA).

### Xenograft transplantations, tissue handling, and immunohistochemistry

Eight-week-old BALB/cA nude mice were intracranially implanted with the indicated cells. Under general anesthesia, either 100 000 TB101 or 50 000 TB107 cells in a total volume of 2 μl of DMEM/F12 were stereotactically injected into the frontal lobe of the mice. Four months after tumor implantation, the mice were euthanized with carbon dioxide. Immediately after the mice were sacrificed, the brains were removed and cut into four coronal sections that were fixed in 4% paraformaldehyde for 24 h and then transferred to 70% ethanol. Thereafter, the fixed tissues were embedded in paraffin. The paraffin blocks were sliced into 4-µm sections on a microtome and then mounted onto poly-L-lysine-coated glass slides. The tissue sections were baked for 2 h at 60 °C, de-waxed in xylene, rehydrated with distilled water, and subjected to heat-induced epitope retrieval using a citrate buffer at pH 6.0 [Dako, Glostrup, Denmark (now Agilent Technologies)] followed by incubation with anti-vimentin antibody (1:100) overnight at 4 °C. The vimentin-bound antibodies were visualized using an EnVision kit (Dako).

### Antibodies

The polyclonal antibody mLrig1-125 against the synthetic peptide (NH2–) CPQPVPRDSGQPGTA (–CONH2) (single-letter amino acid code), which corresponds to the cytosolic C-terminus of mouse Lrig1, was raised in a rabbit. The resulting antiserum was affinity-purified as previously described^36^. The antibody was produced in collaboration with Agrisera AB (Vännäs, Sweden). The polyclonal rabbit anti-human LRIG1-151 antibody has been previously described^35^. The following antibodies were obtained from the corresponding vendors: anti-actin (ab3280; Abcam, Cambridge, UK); anti-actin (clone C4) and anti-p-EGFR (Tyr1086) (Millipore AB, Solna, Sweden); anti-EGFR and anti-MET (C-12; Santa Cruz Biotechnology, Inc., Heidelberg, Germany); anti-p-MET (Tyr1234/1235; D26; Cell Signaling Technology, BioNordika Sweden AB, Stockholm, Sweden); anti-vimentin (clone V9; Dako); and HRP-conjugated donkey anti-rabbit IgG and sheep anti-mouse IgG (GE Healthcare).

### Plasmids

The plasmids *pLVX-TRE3G* and *pLVX-Tet3G* were purchased from BioNordika Sweden AB; *pLVX-LRIG1* has been previously described^37^; and *pLVX-LRIG1-TRE3G* was constructed by ligating PCR-amplified *LRIG1* into a BamHI-MluI-digested *pLVX-TRE3G* vector using an In-fusion HD Cloning Kit (BioNordika Sweden AB). The integrity of all the vector constructs was confirmed with DNA sequencing.

### Gene transduction

To produce lentiviral particles, Lenti-X 293 cells were co-transfected with the relevant expression vectors and the Lenti-X HTX packaging mix using Xfect transfection reagent (Clontech Laboratories) according to the manufacturer’s instructions. Glioma cells were transduced with lentiviral particles according to the manufacturer’s protocol. The cells transduced with either *pLVX-LRIG1* or *pLVX-Puro* were selected with 1 μg/ml puromycin. Cells that had been co-transduced with *pLVX-LRIG1-TRE3G* and *pLVX-Tet3G* were selected with 1 μg/ml puromycin and 1 mg/ml G418. When indicated, LRIG1 expression was induced in the *pLVX-LRIG1-TRE3G*/*pLVX-Tet3G* co-transduced cell lines by the addition of 1 μg/ml doxycycline to the cell culture medium.

### Migration assay

Cell migration was analyzed using a modified Boyden chamber assay. The cells were incubated in the presence or absence of 1 μg/ml doxycycline for 48 h and then plated in the upper chamber of transwell inserts with a pore size of 8 µm (Corning B.V. Life Sciences, Amsterdam, The Netherlands or Becton Dickinson AB, Stockholm, Sweden) in serum-free medium (Thermo Fisher Scientific) in the presence or absence of 1 μg/ml doxycycline. The lower chamber contained medium supplemented with 5% FBS either with or without 1 μg/ml doxycycline. In some experiments, the MET kinase-specific inhibitor PHA-665752 (500 nM final) was added to the upper and lower chambers. After 24 h, the transwell inserts were fixed and stained with 0.1% crystal violet, and the migratory cells were counted in 5 or 10 microscopic fields using a 20× or 40× objective.

### Statistical analyses

Statistical analyses were performed using SPSS 22 software (IBM Corporation, Armonk, NY, USA). ANOVA was used to compare the means of multiple groups. The significance level was set to *p* < 0.05.
